# Psychiatric manifestations of Kleefstra syndrome: a case report

**DOI:** 10.3389/fpsyt.2023.1174195

**Published:** 2023-07-27

**Authors:** Kazunari Yoshida, Daniel J. Müller, Pushpal Desarkar

**Affiliations:** ^1^Azrieli Adult Neurodevelopmental Centre, Centre for Addiction and Mental Health, Toronto, ON, Canada; ^2^Tanenbaum Centre for Pharmacogenetics, Campbell Family Mental Health Research Institute, Centre for Addiction and Mental Health, Toronto, ON, Canada; ^3^Department of Neuropsychiatry, Keio University School of Medicine, Tokyo, Japan; ^4^Department of Psychiatry, University of Toronto, Toronto, ON, Canada; ^5^Institute of Medical Science, University of Toronto, Toronto, ON, Canada; ^6^Department of Psychiatry, Psychosomatics and Psychotherapy, Center of Mental Health, University Hospital of Würzburg, Würzburg, Germany; ^7^Adult Neurodevelopmental Services, Centre for Addiction and Mental Health, Toronto, ON, Canada

**Keywords:** autism spectrum disorder, case report, intellectual disability, Kleefstra syndrome, psychiatric manifestations

## Abstract

**Background:**

Kleefstra syndrome is a rare genetic condition, which affects at least 1 in 120,000 individuals who have a neurodevelopmental disorder, characterized by the core clinical phenotype of intellectual disability, hypotonia, severe speech delay, and distinct facial characteristics with additional clinical features including sleep disturbance, overweight, psychiatric disorders, and autism spectrum disorder. To date, a limited number of case reports of Kleefstra syndrome with psychiatric manifestations have been reported.

**Case presentation:**

We reported a case of a 35-year-old male diagnosed with Kleefstra syndrome, who also had diagnoses of autism spectrum disorder and moderate to severe intellectual disability. He exhibited various psychiatric manifestations, including temporarily manic-like symptoms, excessive eating/overweight, addictive/gambling behaviors, inappropriate and unsafe internet use, sleep disturbance, rigid routines, and behaviors that challenged in the form of meltdowns. These symptoms were eventually relatively successfully managed with a combination of non-pharmacological and pharmacological treatments.

**Conclusion:**

To our knowledge, there is only a limited number of case reports that detail patients with Kleefstra syndrome exhibiting various psychiatric manifestations. Our report adds further knowledge to the paucity of literature and highlights the effectiveness of a combination of non-pharmacological and pharmacological treatments for behavioral/psychiatric difficulties in Kleefstra syndrome.

## Background

Kleefstra syndrome (KS) (OMIM#610253) is a rare genetic condition affecting at least 1 in 120,000 individuals who have a neurodevelopmental disorder and is caused by a mutation in euchromatin histone lysine methyltransferase 1 (*EHMT1*) gene or the deletion of a specific region of chromosome 9 that includes the *EHMT1* gene (1–3). Kleefstra syndrome is characterized by the core clinical phenotype of intellectual disability (ID), childhood hypotonia, severe expressive speech delay, and distinct facial features including microcephaly, brachycephaly, hypertelorism, synophrys, midface hypoplasia, protruding tongue, eversion of the lower lip, and prognathism (4). Additional clinical features include congenital heart and urogenital defects, epilepsy, behavioral problems, sleep disturbance, and overweight (5, 6), as well as psychiatric disorders and autism spectrum disorder (ASD) (5, 7). To date, a limited number of case reports of Kleefstra syndrome with psychiatric manifestations have been reported (8–11). Herein, we report a case of a patient with Kleefstra syndrome exhibiting various psychiatric manifestations and relatively successfully managed with a combination of non-pharmacological and pharmacological treatments. This case study can add to the limited literature on psychiatric manifestations in Kleefstra syndrome and highlight the potential benefits of a combined non-pharmacological and pharmacological approach for addressing these symptoms.

## Case presentation

The patient is a 35-year-old male of Jewish-multi-generational ethnicity (North American) diagnosed with ASD and moderate to severe ID (Full Scale Intellectual Quotient at the 0.1st percentile in the Wechsler Adult Intelligence Scale–Forth Edition (12) at the age of 29), who exhibited various psychiatric manifestations. He is the middle of three siblings. He was delivered by C-section, at 36 weeks, with a fully duplicate urethra, mild pulmonary stenosis, and hypotonia, upon being found to be in distress following a complete cessation of movement for almost 24 h after an otherwise uneventful pregnancy characterized by substantial fetal activity. He was assessed by a geneticist immediately post-birth, at approximately 13 months of age, and at least once prior to reaching 5 years old; during this period, he underwent multiple genetic tests for Down syndrome and mosaicism owing to the presence of suggestive facial features, yet no definitive diagnosis, including Down syndrome, was established. He had a global developmental delay and he received intensive speech therapy, physiotherapy, and occupational therapy to overcome the challenges in his speech and language skills as well as his fine and gross motor skills. He attended special education classes throughout schooling from Grade 1 to high school and obtained a high school diploma. He attended a college vocational program and graduated with a certificate. He has had stable, relatively long-term part-time employment, first with a mailing and printing company for over 8 years, and currently with a company that offers courier services delivered via public transit, also for over 8 years. He had been followed by a cardiologist for his mild pulmonary stenosis. He was also followed by a urologist for his fully duplicate urethra, but his kidneys were determined to be functioning normally around the age of 5. He had a history of surgery for strabismus, the first time on both eyes, the second time on one, and a hearing loss of 10%??−15% in his left ear, which could be features of Kleefstra syndrome. His family history showed that his paternal great-grandmother and paternal great-uncle had bipolar disorder, whereas his mother had a mood disorder unspecified. One of his paternal aunts demonstrated developmental delays in speech and walking, and exhibited mild autistic tendencies such as difficulty in social interactions, although she was not formally diagnosed with ID or ASD.

The patient's behavior at home from the time he was a toddler was inconsistent and frequently extremely challenging. He would destroy toys, shred books, throw tantrums, and damage walls by head-banging. His school behavior, in contrast, was generally compliant. There was a major change in his behavior at the age of 9, which occurred concurrently with a shift in his educational environment, including alterations in school and teacher, as well as the departure of his live-in nanny who had been present since he was 3 years old. He initially seemed to adjust well to a change of schools in the fourth grade. His behavior became aggressive and unmanageable after about 10 weeks, similar to that at home. The pediatric neurologist who had followed the patient since he was 12 months old helped them obtain a referral to a psychiatrist, who then followed the patient from the age of 9 to the age of 26. The psychiatrist diagnosed the patient with ASD on the first visit at the age of 9. At the age of 9, he was also diagnosed with moderate to severe ID. The patient received pharmacological treatment, including propranolol and clonidine, and then haloperidol and risperidone to treat his behaviors that challenge, many of which were likely driven by anxiety. Propranolol and clonidine were discontinued because they induced lethargy and significant weight gain. Haloperidol was replaced by risperidone because of concerns about the possibility of tardive dyskinesia. Eventually, the dosage of risperidone was reduced by one-third to 1 mg and subsequently by half to 0.5 mg, and a reduced dosage of haloperidol (0.5 mg) was reintroduced due to the persistent weight gain associated with risperidone. During the course of treatment, attempts were made to titrate and discontinue these medications; however, there was a significant increase in the patient's anxiety. Consequently, these dosages were continued until the age of 28.

Further significant changes in the patient's behavior were observed during an 18-month period, when he was 27–28 years old. When he turned 27, the patient's younger sister got married and older brother got engaged. This led him to conclude that he, too, should get married. This resulted in numerous incidences of impulsivity and inappropriate verbal social media activity. A relationship was established with an occupational therapist to help him deal with these issues, and the situation settled down for a while. Approximately 1 year later, he exhibited a resurgence in his markedly impulsive behavior, which was characterized by extreme talkativeness, hasty and unsuitable marriage proposals, and episodes of binge-eating. This period culminated in the most extreme manic-like episode around the time of the Jewish holiday Shavuot that commemorates the giving of the Torah on Mount Sinai. The patient had regularly been participating for a number of years, on his own accord, in the custom to remain awake all night on the first night of Shavuot to engage in Torah learning. It was noted, a month before the holiday, that he looked as if he had not slept in days, and that he appeared to have lost the ability to behave courteously in public. The patient appeared to be sleeping poorly in the days leading up to Shavuot, and he was clearly exhausted. That year, the two-day holiday of Shavuot immediately followed the Sabbath day, so the patient, who was traditionally observant, spent a lot of time in synagogue over three consecutive days, attending long morning services and shorter evening services. On the first day of the holiday following morning services after the all-night learning session, he did not return home until about 6:45 p.m., after a 26-h absence. Following the long period of non-sleep, instead of returning home, he had spent the entire afternoon visiting numerous friends and acquaintances over a widespread area spontaneously without prior arrangement. Around that time period, he was talking even more rapidly and loudly. His family, therapist, and co-workers all noticed that he was more anxious, more impulsive, and more high-energy than normal. His family physician was of the opinion that most of his extreme behavior was caused by sleep deprivation. He advised the patient that proper sleep was of the utmost importance.

As part of his family physician's assessment the day after the holiday, a blood test was within normal limits, except for an elevated WBC of 13.5/L and the presence of ketones (0.5 mmol/L) and protein (1.0 g/L) in the urine. Two weeks later, the blood work and urinalysis were normal. He had been taking risperidone 0.5 mg and haloperidol 0.5 mg for 3 years until that visit with his family physician. The risperidone dose was increased from 0.5 to 0.75 mg, and his symptoms improved the following day. The total length of the episode was 5 days, and all symptoms improved after 1 day of increased risperidone dosage and a few nights of improved sleep. No prior history of prolonged low mood or depression and no marked changes in his mood was observed during this episode. He was neither irritable nor euphoric and was not observed to be emotionally labile. He had no overt delusional beliefs, grandiosity, flight of ideas, or unrestrained buying sprees. During this period, he underwent additional genetic testing, supplementing previous assessments between the ages of 4 and 9 that did not result in a Fragile X or Prader-Willi diagnosis. Microarray analysis yielded negative results; however, subsequent testing for X-linked intellectual disability identified two minor translocations of unknown significance, also observed in his mother and older brother, which were later classified as benign. After that, when he was referred for a psychiatric consultation (i.e., diagnostic clarification and medication recommendations) in an urban, tertiary-level mental health care facility at the age of 29, no indication of anxiety disorders, obsessive-compulsive disorder, or psychotic disorders was noted. His symptoms without abnormally and persistently elevated, expansive, or irritable mood (i.e., a discrete episode of significantly diminished sleep for several days, even when compared to his typical sleep pattern that yields minimal rapid eye movement (REM) sleep, frequently accompanied by numerous episodes of sleep apnea, psychomotor agitation, and increased energy in the context of the Jewish holiday Shavuot) were resolved within only 1 day of increased risperidone at a very low dose of 0.75 mg and his usual 0.5 mg dose of haloperidol. The resolution of symptoms occurred much faster and in response to an inadequate dose of antipsychotic than would be expected for a “true” hypomanic or manic episode. Therefore, this short period of symptoms was not in keeping with a hypomanic or manic episode and was therefore incompatible with the diagnosis of bipolar disorder. The constellation of symptoms and behaviors observed was considered as an exacerbation of his usual behaviors in the context of triggers or stressors, which was frequently observed in individuals with ASD. For pharmacological treatment, the same medications (i.e., risperidone 0.75 mg and haloperidol 0.5 mg) were continued.

After the episode mentioned above, he was followed by his family physician and received the same medications (i.e., risperidone 0.75 mg and haloperidol 0.5 mg) until he was 32 years old. He was referred to the same psychiatrist in the tertiary-level mental health care facility again at the age of 32 because of a number of presenting concerns that were significantly impacting his ability to participate successfully and safely in his community. The main behaviors of concern included (1) excessive eating and inappropriate access to food items; (2) use of internet/social media/online dating platforms and gambling concerns; (3) rigid routines and meltdowns; and (4) sleeping concerns. More specifically, he had been eating excessive amounts of food. His weight thus fluctuated a lot and had reached as high as 120 kg. He had also been engaged in inappropriate behaviors in accessing baked goods and other proscribed foods, including panhandling on the subway. He also used to purchase scratch-and-win tickets and would sometimes spend between $80 and $100 per day on them. In addition, he was regularly using the internet and various apps, texting and emailing people outside of appropriate hours, and sending sexually suggestive messages to co-workers and acquaintances. He also engaged in the exchange of personal information through dating apps, putting his financial and personal safety at risk. This behavior had been observed for about 5 years. However, significant concerns were raised over the risk to his financial and personal safety in the 6–7 months preceding the referral to the psychiatrist at the age of 32. He also had a lot of established routines, which he had followed stringently for a long time. For instance, he needed to have his phone fully charged at all times. Any deviation from his routine, such as a phone battery level of 60%, could result in a meltdown. These meltdowns involved yelling, crying, and, in rare cases, physical aggression directed against a family member. With regards to his sleep quality, the amount of sleep he got was very short (i.e., 2–5 h per night). He was prescribed melatonin 10 mg in the past, but it was ineffective. He was independent with most of his activities of daily living, although he required verbal reminders to complete many of these tasks. He was receiving support from a private occupational therapist and support worker. A blood test, including a complete blood count, an ionogram, liver function, and kidney function were all normal at the age of 32, except for a slightly high total bilirubin level (27 μmol/L). The psychiatric consultation interpreted these behaviors as understood in the context of ASD and ID at that time. During the consultation period at the age of 33, he received the results of? the whole genome sequencing project he participated in around the age of 30 and received a diagnosis of Kleefstra syndrome with a variant in the *EHMT1* gene. More specifically, he was found heterozygous for the c.546dupG variant in the *EHMT1* gene. His parents also received genetic testing, and it was found that his variant was de novo. His head MRI at the age of 32 showed no abnormalities. At the age of 33, a chest X-ray performed was normal. No blockages in the pulmonary artery were noted. An echocardiogram showed very mild aortic regurgitation, but cardiac function was otherwise normal.

During his consultation by the same psychiatrist in the tertiary-level mental health care facility between the ages of 32 and 35, he was referred to a metabolic clinic and a problem gambling and technology use clinic and underwent behavioral therapy (BT) for his excessive eating and inappropriate access to food items, use of internet/social media/online dating platforms and gambling concerns, rigid routines, and meltdowns. He received pharmacological treatment with metformin as well as diet and exercise interventions at the metabolic clinic. He underwent a series of group therapy sessions for gambling and technology use weekly or bi-weekly for 10 months at the problem gambling and technology use clinic and BT at 1- to 4-week intervals for 12 months. During the BT, he kept a journal to track his food intake, reporting his triggers and temptations for the use of dating platforms and gambling, and he tracked his emotions using a visual emotion-tracking sheet. He also continued taking risperidone 0.75 mg and haloperidol 0.5 mg. He eventually and cumulatively lost approximately 35 kg while on metformin 2,000 mg as well as through diet and exercise interventions over approximately 2 years after attending the metabolic clinic. He successfully completed the therapeutic intervention program at the problem gambling and technology use clinic and the BT. His psychiatric condition and behaviors (i.e., excessive eating and inappropriate access to food items, use of internet/social media/online dating platforms and gambling concerns, and rigid routines and meltdowns) mostly stabilized for a time with these treatments without adverse effects, even after he experienced an environmental change (i.e., moving to a new apartment). For his sleep disturbance, he also attended a sleep clinic consultation and underwent polysomnography and actigraphy assessments. Compared with the results he received around the age of 28, his apnea-hypopnea index mildly increased overall and moderately increased during REM sleep with a minimum oxygen desaturation of 84%. The need for preventive management of sleep disturbance was discussed because of its likely detrimental effects on the mood and behaviors of individuals with Kleefstra syndrome, especially the risks of regression. For pharmacological treatment, he first received trazodone 50 mg and clonazepam 1 mg, as required, in addition to the transition of the timing of risperidone 0.75 mg and haloperidol 0.5 mg from the morning to the evening. He continued to take 50 mg trazodone nightly at bedtime. His sleep problems were eventually managed with 1 mg clonazepam as required at times of extraordinary sleep disruption. Finally, he was discharged from the clinical consultation by the attending psychiatrist. His father, who was a caregiver and substitute decision maker, reported that the patient's symptoms mentioned above were relatively managed with a combination of non-pharmacological and pharmacological treatments. The timeline of the patient's psychiatric/medical and treatment history was summarized in Table 1.

**Table 1:** Timeline of the patient's psychiatric/medical and treatment history.

**Age**	**Brief psychiatric and medical history**	**Treatments and assessments**
Birth	Born, delivered by C-section in distress at 36 weeks, with fully duplicate urethra, mild pulmonary stenosis, hypotonia	Followed by urologist and cardiologist; genetics consultation
Early childhood	Global developmental delay	Speech therapy, physiotherapy, occupational therapy; infant stimulation/nursery program with frequent diagnostic assessments and therapies. Followed by neurologist; further genetics consultations
9 years old	Diagnosed with ASD and mild to moderate ID	The patient underwent pharmacological treatment for anxiety-driven behaviors that challenge, including propranolol, clonidine, haloperidol, and risperidone, with adjustments made due to side effects such as lethargy, weight gain, and concerns about tardive dyskinesia. Propranolol and clonidine were discontinued because they induced lethargy and significant weight gain. Despite attempts to titrate and discontinue haloperidol and risperidone, increased anxiety necessitated the continuation of adjusted dosages (i.e., 0.5 mg each) until the age of 28.
27–28 years old	At ages 27–28, the patient had significant behavioral changes (e.g., numerous incidences of impulsivity and inappropriate verbal social media activity), triggered by his younger sister's marriage and older brother's engagement, which led him to seek marriage. Despite temporary stability achieved through occupational therapy, he later exhibited a resurgence of impulsive behavior, culminating in an extreme manic-like episode around the Jewish holiday Shavuot.	Twice-weekly OT. Further genetic testing. Risperidone dose increased from 0.5 to 0.75 mg
29 years old	A psychiatric consultation, involving diagnostic clarification and medication recommendations, was conducted at an urban, tertiary-level mental health care facility. The assessment determined that the manic-like episode experienced by the patient was an exacerbation of their typical behaviors in response to triggers or stressors. This is frequently observed in individuals with ASD.	Continued risperidone 0.75 mg and haloperidol 0.5 mg
33 years old	Diagnosis of Kleefstra Syndrome (EHMT1 gene variant)	Followed by a geneticist
32–35 years old	The main behaviors of concern included (1) excessive eating and inappropriate access to food items; (2) use of internet/social media/online dating platforms and gambling concerns; (3) rigid routines and meltdowns; and (4) sleeping concerns. His excessive eating and inappropriate access to food items could be mainly understood in the context of ID and/or ASD. His ASD could contribute to his addictive/gambling behaviors and inappropriate/unsafe internet use. His rigid routines and meltdowns could be understood in the context of ASD and/or ID. Given the lack of indication of mood disorders (e.g., major depressive disorder and bipolar disorder) and no other remarkable environmental factors contributing to sleep disturbance, his sleep disturbance was understood as a clinical manifestation of Kleefstra syndrome.	(1) Behavioral therapy and referral to a metabolic clinic (metformin 2,000 mg as well as diet and exercise interventions; (2) referral to problem gambling and technology use clinic; (3) behavioral therapy and continuation of pharmacological treatment (risperidone 0.75 mg and haloperidol 0.5 mg); and (4) sleep clinic consultation and pharmacological treatment (e.g., transition of risperidone 0.75 mg and haloperidol 0.5 mg timing from morning to evening and clonazepam 1 mg as required)

ASD, autism spectrum disorder; EHMT1, euchromatin histone lysine methyltransferase 1; ID, intellectual disability; NA, not applicable; OT, occupational therapy.

## Discussion and conclusions

In this report, we presented a case of a patient with Kleefstra syndrome, resulting from a c.546dupG mutation in exon 3 of the EHMT1 gene (NM_024757.4), who exhibited multiple psychiatric manifestations, thereby enriching the limited body of literature on this rare condition. The patient exhibited temporarily manic-like symptoms, excessive eating/overweight, addictive/gambling behaviors, inappropriate and unsafe internet use, sleep disturbance, rigid routines, and behaviors that challenged in the form of meltdowns, which were eventually relatively successfully managed with a combination of non-pharmacological and pharmacological treatments.

It has been reported that behavioral/psychiatric problems are exhibited by 73% of individuals with an intragenic *EHMT1* mutation (5). In this regard, the presence of behavioral/psychiatric symptoms in the present case is consistent with the clinical features of Kleefstra syndrome. This case also had several clinical manifestations commonly observed in Kleefstra syndrome, including congenital heart and urogenital defects, sleep disturbance, and overweight, as well as ID and ASD (5–7, 11). Given the lack of indication of mood disorders (e.g., major depressive disorder and bipolar disorder) and no other remarkable environmental factors contributing to sleep disturbance, his sleep disturbance was understood as a clinical manifestation of Kleefstra syndrome. A previous case report highlighted that sleep disturbances in Kleefstra syndrome might be followed by rapid regression and the importance of prompt treatment of sleep disturbances with medications to halt further regression (11). In our case, his preceding sleep disturbances might be one possible explanation for his behaviors that challenged, which were observed in the absence of mania/hypomania. Prompt management of sleep disturbances, including an increase in risperidone dosage up to 0.75 mg (initially prescribed to manage behaviors that challenge), or use of clonazepam 0.5 or 1 mg as required, might prevent further behavioral regression in this patient. His excessive eating and inappropriate access to food items could be mainly understood in the context of ID and/or ASD, although haloperidol and risperidone, despite being taken as low doses, may have partly contributed to his excessive eating. To the best of our knowledge, this is the first case example of Kleefstra syndrome with multiple psychiatric manifestations including addictive/gambling behaviors and inappropriate/unsafe internet use. His ASD may be a contributing factor in his addictive/gambling behaviors and inappropriate/unsafe internet use, given the correlation between problematic internet use and gaming disorder that has been reported in individuals with ASD (13). Nevertheless, the complexity and difficulty of the psychopathological assessment in people with ID should be noted, such as diagnostic overshadowing and difficulty of the specifications of quality and duration of symptoms (14). For instance, it is important to ascertain the presence or absence of bipolar disorder in individuals with Kleefstra syndrome who exhibit psychomotor agitation, decreased sleep, mood fluctuations, and aggression, as bipolar disorder is frequently comorbid with ID and/or ASD, although often not adequately identified in clinical practice (15).

Various psychiatric manifestations of the patient were reasonably successfully managed with a combination of non-pharmacological and pharmacological treatments in the context of ID and ASD. The UK National Institute for Health and Clinical Experience (NICE) guidelines suggest non-pharmacological treatment, including a psychosocial intervention, for behaviors that challenge in adults with ID and/or ASD as the first-line treatment (16, 17). In addition, when antipsychotic medications are used for the treatment of behaviors that challenge in adults with ID and/or ID, the guidelines also recommend they are used in combination with psychological or other interventions (16, 17). Thus, consistent with the treatment principle, the treatment strategy in our case combined non-pharmacological treatments, including BT and occupational therapy, and antipsychotic medications. However, notably, research and evidence on the pharmacological treatment of behaviors that challenge in adults with ID and/or ASD are still limited. Therefore, clinicians will be encouraged to use psychotropic medications with caution in individuals with Kleefstra syndrome who can present ID and/or ASD as clinical features. Furthermore, his other clinical manifestations (i.e., excessive eating/overweight, addictive/gambling behaviors, inappropriate/unsafe internet use, and sleep disturbance) were also managed with a combination of non-pharmacological and pharmacological treatments. Although the effectiveness of these therapies has been demonstrated in general (18, 19), to the best of our knowledge, there is still no literature that investigates the effectiveness of these therapies in individuals with Kleefstra syndrome. Given that, our important findings provide essential insight that will lead to the development of more effective treatment plans to address behavioral and psychiatric symptoms of Kleefstra syndrome.

In conclusion, our case report describes an example of various psychiatric manifestations and the successful combination of non-pharmacological and pharmacological treatments in Kleefstra syndrome. Given very limited reports of Kleefstra syndrome in the literature, especially cases with psychiatric manifestations (8–11), our case report will add further knowledge to the paucity of literature on this rare genetic condition and underscore the importance of a combination of non-pharmacological and pharmacological treatments for behavioral/psychiatric symptoms in Kleefstra syndrome, including behaviors that challenge.

## Data availability statement

The original contributions presented in the study are included in the article/supplementary material, further inquiries can be directed to the corresponding author.

## Ethics statement

Written informed consent for any potentially identifiable data included in this Case Report was obtained by the participant's father, who is a substitute decision maker.

## Author contributions

KY and PD did the literature search and wrote the first draft of the manuscript. All authors wrote the report and approved the final version of the manuscript.
